# The Impact of Advertising Image Types on Consumer Purchasing Behavior of Fresh Agricultural Products

**DOI:** 10.3390/foods14223915

**Published:** 2025-11-15

**Authors:** Fan Huang, Yumeng Gu, Zhonghu Bai, Yani Dong

**Affiliations:** 1 College of Economics and Management, Huazhong Agriculture University, Wuhan 430070, China; 2 Institute of Finance and Economics, Wuhan City Polytechnic, Wuhan 430064, China; 3 Business School, Hubei University of Economics, Wuhan 430070, China

**Keywords:** advertising images, guilt, self-construal, purchase intention

## Abstract

Advertising images constitute an important factor influencing consumer purchase intentions in commercial settings. Drawing on the perspective of self-conscious emotions, this study examines the impact of advertising image types for fresh agricultural products on consumer purchase intentions and explores the underlying mechanisms. Advertising images are classified into three categories: meat-typical, animal-typical, and composite. Evidence from two randomized experiments reveals the following findings: (a) The effectiveness of the three advertising image types in promoting purchase intentions follows the order of meat-typical > animal-typical > composite; (b) guilt mediates the relationship between advertising image types and purchase intentions, such that composite images evoke greater guilt than meat-typical and animal-typical images, thereby reducing consumer willingness to purchase; and (c) self-construal partially moderates the mediating effect of guilt, in that interdependent self-construal consumers exposed to composite advertising images are more likely to experience heightened guilt and consequently exhibit lower purchase intentions. This study extends the application of animal-related classifications in advertising and marketing research and provides new empirical evidence and practical insights for the design of advertising strategies for fresh agricultural products.

## 1. Introduction

In most countries, more than 90% of the population consumes meat regularly [1]. Although meat consumption is widespread globally, it has long been associated with the “meat paradox,” which refers to the psychological conflict between the enjoyment of meat and moral concern over animal welfare [2]. With the increasing prevalence of meat advertising in market communications, consumers are frequently exposed to various images of fresh meat. In particular, advertisements that emphasize animal characteristics may heighten consumers’ awareness of animals, thereby eliciting stronger feelings of guilt [3,4] and negatively influencing their consumption behavior.

Although advertising plays an important role in shaping consumer attitudes, existing studies have primarily examined advertising techniques—such as anthropomorphism and visual design elements—and the positive emotional responses they evoke [5,6,7,8,9]. However, variations in advertising content and the potential negative emotional mechanisms they elicit have received less attention. In particular, in the context of meat advertising, limited research has explored how different image types (e.g., meat-typical, animal-typical, and composite images) influence consumers’ emotions and behaviors through the salience of animal features. Accordingly, this study addresses a central question: How do different types of meat advertising images influence consumers’ purchase intentions?

Previous research has identified guilt as a key self-conscious emotion in meat consumption contexts, reflecting individuals’ moral evaluation of the harm caused to animals by their own behavior [10]. When advertising images make the connection between animals and food more salient, consumers are more likely to experience guilt, which in turn reduces their purchase intentions [11,12]. Compared with other emotions, guilt is not only closely linked to moral judgment but also serves a regulatory and self-corrective function in consumer decision-making. However, few studies have focused on the different degrees of guilt experience caused by different categories of advertising content, which in turn affects the willingness to purchase. Therefore, investigating how different types of advertising images influence consumer behavior by eliciting guilt is crucial for understanding the psychological mechanisms underlying the “meat paradox.”

The study explores how various types of advertising images impact consumers’ intentions to purchase meat, utilizing the theory of self-conscious emotions and the concept of self-construal. It also examines the underlying mechanisms and boundary conditions involved in this relationship. Following the classification of animal characteristics proposed by Kubberød et al. [13], meat advertisements were categorized into three types—meat-typical, animal-typical, and composite images—to examine differences in animal salience and their corresponding psychological effects from the perspective of advertising content. Furthermore, the study examined the mediating role of guilt to clarify how animal salience influences consumers’ purchase decisions by eliciting guilt. In addition, self-construal was introduced as a moderating variable to explore how this mechanism differs between independent and interdependent cultural orientations.

To test the proposed hypotheses, we conducted two web-based experiments. Experiment 1 used pork advertisements as stimulus materials to test the impact of three types of advertising images (typical meat, typical animals, and composite images) on consumers’ willingness to buy and tested the mediating role of guilt. Experiment 2 used beef advertisements to further validate the proposed mechanism and examined the moderating effect of self-construal on the relationship among advertising image type, guilt, and purchase intention. The robustness and applicability of the findings across different categories were established through the two experimental settings, which included pork in Experiment 1 and beef in Experiment 2. The findings offer theoretical guidance and practical implications for meat advertising design and ethical marketing practices.

## 2. Literature Review and Research Hypothesis

### 2.1. Elaboration Likelihood Model

The Elaboration Likelihood Model (ELM) posits that individuals process persuasive information through two distinct routes: the central route and the peripheral route. When individuals have high motivation and cognitive ability, they tend to follow the central route to conduct in-depth analysis and evaluation of the information content; when motivation or ability is insufficient, they are more likely to rely on peripheral cues, such as relying on surface features, such as images, packaging, colors, or celebrity endorsements, to make judgments [14,15]. In low-involvement or daily purchase situations, consumers’ information processing is often constrained by time and cognitive resources, leading them to rely more heavily on peripheral visual cues when forming attitudes [16,17]. Studies have shown that in the field of fresh food, visual cues are the main source of information for consumers to obtain product quality and freshness [18,19]. Therefore, in advertising, different types of images may evoke distinct emotional and evaluative responses through variations in visual salience. For instance, advertisements featuring meat-only images, animal-only images, or composite images combining both may guide consumers’ processing along the peripheral route.

Building on this framework, the present study posits that the content of meat advertising images influences consumers’ visual attention and emotional responses, and may shape purchase intentions by eliciting varying degrees of moral emotion—particularly guilt—through the peripheral processing route.

### 2.2. The Influence of Advertising Images on Purchase Intention

In marketing practice, advertising images of fresh agricultural products generally appear in three forms. For example, the packaging of pork products by Chinese enterprises such as CP Foods and Shuanghui can be broadly categorized into three types: advertising images that display only pork slices, those that display only the pig itself, and composite images that simultaneously display both pork slices and the pig. Kubberød et al. classified animal characteristics into meat typicality, animal typicality, and personification [13]. Meat typicality refers to red meat associated with blood, where blood itself represents a highly stimulating visual cue [20]. Animal typicality refers to meat or meat products that have not been deeply processed and remain strongly connected to the animal body, thereby not concealing their animal nature; for instance, animal organs are closely linked to live animals and often evoke associations with them [13,21,22]. Personification refers to animal images that are emotionally closer to humans, such as pets. Drawing on Kubberød et al.’s classification of animality, this study further categorizes advertising images of fresh agricultural products into three types: meat-typical images displaying only red meat slices, animal-typical images displaying only animal figures, and composite images simultaneously presenting red meat slices and animal figures.

Research has shown that advertising images are one of the key carriers of information dissemination, capable of conveying product-related information to consumers [23]. Images in advertisements are not only a process of visual psychology and cognitive transformation but also exert direct visual stimulation on consumers through visualized symbols, while guiding their attention via informational cues to achieve the transformation from vision to perception and from image to information, thereby facilitating the dissemination of marketing content [24]. Previous studies have found that the visual appeal of advertising images can quickly trigger consumers’ psychological perceptions, evoke positive emotional responses [25], and consequently enhance their purchase intentions [26]. Therefore, companies often employ a variety of advertising images in their marketing practices.

As fresh meat products are typical fast-moving consumer goods, consumers are often subject to time and attention constraints when exposed to related advertisements, leading to relatively superficial processing of advertising information. According to the Elaboration Likelihood Model (ELM), when individuals have low motivation or cognitive ability, persuasion tends to occur via the peripheral route, relying primarily on perceptual and visual cues rather than detailed information processing [14,15]. In low-involvement purchasing situations, such as buying fresh products, consumers are therefore more likely to rely on peripheral cues such as images, colors, and packaging design to form initial impressions of products [18,19]. Based on this, we posit that different forms of image presentation in advertisements may evoke distinct emotional responses and evaluative tendencies by altering visual salience. Specifically, when advertisements feature meat-typical images, consumers’ attention is primarily directed toward the processed meat itself, psychologically distancing the “animal” from the “food.” This distancing may reduce negative emotions associated with animal harm, thereby enhancing product evaluation and purchase intention.

When advertisements depict animals in their typical form, they explicitly remind consumers that meat products originate from the body parts of living animals [27,28]. This reminder evokes the “meat paradox,” wherein people enjoy eating meat but simultaneously reject harming sentient animals [2,29]. When advertisements feature animal-typical imagery, consumers find it more difficult to suppress moral concerns about animal welfare, thereby decreasing their purchase intentions. Moreover, prior research has shown that unprocessed meat is more likely to elicit empathy toward animal slaughter than processed meat, further reducing consumers’ willingness to eat meat [3]. Consequently, animal-typical advertising images are more likely to heighten consumers’ awareness of animal suffering associated with meat consumption, thereby diminishing purchase intention.

When advertisements combine both meat-typical and animal-typical elements in composite images, the connection between animals and food becomes more salient, eliciting stronger feelings of guilt and further suppressing purchase intention [11,12]. Based on this, we propose the following hypothesis:

**Hypothesis 1** **(H1).** *Meat-typical images are more likely than animal-typical images to enhance consumers’ purchase intentions.*

**Hypothesis 2** **(H2).** *Animal-typical images are more likely than composite images to enhance consumers’ purchase intentions.*

### 2.3. Moral–Emotion Routes: The Guilt Emotion Pathway

Recent research suggests that meat consumption is not merely a nutritional behavior but is often accompanied by moral conflicts related to animal suffering and the ethical implications of animal use. Such conflicts can evoke negative moral emotions in consumers, including disgust, contempt, anger, shame, and guilt. In research on meat consumption, particular attention has been given to emotional responses such as disgust, shame, and guilt [30,31]. Previous studies indicate a certain degree of correlation among disgust, guilt, and shame. Disgust is defined as an aversive, avoidance-oriented response to unpleasant stimuli [32]. It represents a primary and automatic reaction associated with bodily rejection or moral purity. This reaction can activate consumers’ moral self-evaluation, thereby eliciting more complex self-conscious emotions such as shame or guilt [33]. Shame involves the negative evaluation of the self as a whole by others and often leads to avoidance or concealment behaviors. In contrast, guilt focuses on responsibility for specific actions and motivates individuals to make reparations or behavioral corrections.

Furthermore, studies suggest that shame is a more “public” emotion, typically elicited by social exposure or disapproval of one’s flaws or behaviors. In contrast, guilt is regarded as a more “private” emotional experience that arises from internalized moral standards and self-generated pangs of conscience [34]. Based on these distinctions, our study posits that, in the context of meat consumption, guilt represents the primary and more proximal negative emotion influencing consumers’ willingness to consume meat. This is because consumers may perceive their meat-eating behavior as causing harm to animals, thereby motivating them to modify their consumption patterns (e.g., reducing or avoiding meat) to alleviate moral discomfort or guilt associated with eating meat. Accordingly, the following discussion primarily focuses on the mediating role of guilt in the relationship between advertising image type and purchase intention.

### 2.4. The Mediating Role of Guilt

Guilt is a secondary emotion that arises from self-evaluation. Individuals experience guilt when they perceive that their actions have violated moral or behavioral standards they personally value [35]. Research has demonstrated that meat consumption often elicits guilt, as individuals experience moral conflict between satisfying dietary preferences and avoiding harm to animals [11]. Studies on animal-related visual cues indicate that advertisements or labels featuring animal imagery can heighten consumers’ awareness that meat originates from living animals, thereby eliciting moral emotions such as guilt and reducing their willingness to consume meat [36,37]. Therefore, we can predict that when the advertisement shows typical images of animals, consumers will be prompted to evaluate the behavior of “killing animals for eating meat”, which will activate consumers’ moral emotions, thus generating guilt and ultimately affecting consumers’ willingness to buy.

Existing research has demonstrated that cues linking meat to animals can heighten feelings of guilt, thereby decreasing individuals’ willingness to consume meat. For instance, when consumers were simultaneously exposed to images of raw meat and live animals [38,39], the perceived connection between animals and meat became stronger, eliciting greater guilt and subsequently reducing meat consumption. Therefore, when advertisements feature composite images, the association between eating meat and killing animals becomes more salient, intensifying consumers’ feelings of guilt. In contrast, advertisements displaying meat-typical images primarily emphasize the product itself, rendering the animal’s victimized state implicit or less noticeable. As noted earlier, advertisements that depict only meat slices may psychologically distance animals from food, thereby eliciting less guilt and exerting minimal reduction in consumers’ purchase intention.

Cognitive dissonance may occur when individuals recognize that their meat consumption conflicts with their moral beliefs about animal welfare, often manifesting as a negative emotional state associated with guilt [39]. In this state of dissonance, consumers experience a tension between their actions and moral convictions, motivating them to adopt various coping strategies [29,40,41,42]. According to cognitive dissonance theory, individuals can alleviate such tension by adjusting inconsistent cognitions—for instance, by rationalizing the meat paradox. Consequently, to mitigate guilt, consumers may reduce their willingness to consume meat [2]. Accordingly, we propose the following hypothesis:

**Hypothesis 3** **(H3).** *Guilt mediates the relationship between advertising image types and purchase intentions.*

### 2.5. Moderating Effect of Self-Construal

Existing research indicates that the experience of guilt depends not only on individuals’ self-evaluation of their behavior but also on their social–cognitive orientation. According to self-construal theory, differences in self-construal influence individuals’ sensitivity to social norms and situational cues [43]. Individuals with interdependent self-construals place greater emphasis on social relationships and others’ evaluations, tending to assess their behavior through the lens of social and moral norms. Consequently, they are more likely to experience social emotions such as guilt when their actions violate social expectations [44,45]. In contrast, individuals with independent self-construals emphasize personal agency and autonomy and exhibit lower sensitivity to social and moral emotions. In the context of meat advertising, composite images emphasize the connection between animals and food. Interdependent individuals are more likely to recognize that eating meat implies harming animals, thereby eliciting stronger guilt. Conversely, independent individuals tend to focus on product attributes and are less susceptible to moral cues.

Self-construal refers to how individuals perceive and define the relationship between themselves and others, including their understanding of personal thoughts, emotions, and behaviors [46]. Self-construal theory distinguishes between two dimensions: independent self and interdependent self [47]. The independent self emphasises individuality and stability, highlighting internal attributes and autonomy, whereas the interdependent self stresses relationality and interconnectedness with society and others, underscoring the importance of relationships and social background, and others, underscoring the importance of relationships and social background. While individuals may possess both traits, they typically exhibit a stronger inclination towards one orientation [48,49].

Prior research has shown that self-construal types influence individuals’ information processing styles. Individuals with an independent self-construal tend to adopt holistic processing, focusing more on the core features of stimuli while overlooking contextual factors [50,51]. Conversely, individuals with an interdependent self-construal are inclined towards analytic processing, paying greater attention to the associations between objects and contexts [52,53], and are accustomed to integrating information across multiple dimensions [54,55]. Accordingly, individuals with an independent self-construal focus more on product attributes when processing advertising information [56], and their decisions tend to be more rational [57,58], whereas those with an interdependent self-construal are more susceptible to social norms and contextual cues, leading to more affect-driven decision-making [59]. Based on this, when advertisements present composite images (simultaneously depicting meat and animal imagery), individuals with different self-construals exhibit divergent responses. For independent individuals, their rational, product-focused cognitive style makes them more likely to concentrate on the quality of pork itself when exposed to composite images, without actively forming the association that “eating meat = killing animals”, and thus their purchase intentions are not significantly reduced. In contrast, interdependent individuals actively seek and construct contextual associations within advertising information [60]; composite images make the link between meat and animals more salient, thereby reinforcing the association that “eating meat implies killing animals” and eliciting stronger guilt. To alleviate this negative emotion, interdependent individuals tend to reduce their purchase intentions for fresh meat products [2].

Moreover, when advertisements display only meat-typical or animal-typical images, regardless of self-construal type, consumers primarily focus on the focal content of the images and lack the initiative to form the association that “eating meat = killing animals.” In such cases, consumers are more likely to infer meat quality based on product cues, leading to similar purchase decisions. Therefore, differences between self-construal types are primarily manifested in the context of composite advertisements. Accordingly, we postulated the following hypothesis:

**Hypothesis 4** **(H4).** *When consumers exhibit an interdependent (vs. independent) self-construal, their purchase intention for fresh meat products will be significantly lower when exposed to composite (vs. meat-typical/animal-typical) advertising images.*

**Figure 1:**
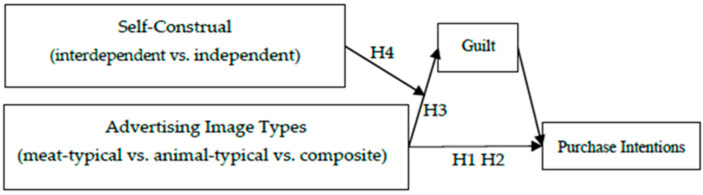
Conceptual model.

The overall conceptual model is depicted in Figure 1.

In recent years, numerous studies on the “meat paradox” have examined the moral conflicts and regulatory mechanisms underlying consumers’ meat consumption behaviors. Research has demonstrated that dietary identity (e.g., vegetarians vs. meat eaters) significantly influences individuals’ moral–emotional responses. Vegetarians typically exhibit stronger moral sensitivity and more negative emotional reactions, whereas meat eaters often alleviate guilt through cognitive distortion. Moreover, factors such as the level of meat attachment, the presence of animal welfare cues, and traceability information have also been found to significantly affect consumers’ moral–emotional responses. For instance, individuals with a high level of meat attachment may deny animals’ perceptual capacities when confronted with the moral conflict of meat consumption, thereby reducing feelings of guilt [61]. When advertisements display information related to animal welfare or traceability labels, consumers’ moral emotions are more easily activated, leading to guilt and subsequently influencing purchase decisions. Although the present study did not directly examine these influencing factors, the ongoing evolution of Chinese consumers’ dietary culture and the fresh food market offers valuable theoretical perspectives and promising directions for future research.

It is important to note that the moral–emotional responses elicited by different animal species (e.g., pigs and cattle) may vary. Previous research has indicated that cattle are considered sacred in certain cultures, and their slaughter tends to evoke stronger moral conflicts [62]. Similarly, some Chinese consumers also perceive cattle as culturally significant or “sacred” animals. However, existing studies on meat consumption in China have generally overlooked the influence of species differences on moral–emotional responses. Drawing on this cultural distinction, the present study incorporated two experimental scenarios—pork and beef—to examine how differences in animal species influence consumers’ emotional responses and purchase intentions. This design not only addresses a gap in the existing literature but also enhances the generalizability and robustness of the study’s conclusions.

## 3. Experiment 1: The Impact of Advertising Image Types on Purchase Intentions and the Mediating Role of Guilt

### 3.1. Experimental Purpose

Experiment 1 used a between-subjects design, dividing participants into three groups based on the type of advertising image: meat-typical, animal-typical, and composite. The goal of this experiment was to examine how different types of advertising images affect purchase intentions in the context of pork consumption and to test the mediating role of guilt. Since this study explores the mechanisms underlying meat-related advertising images, it is important to note that meat, in addition to its high nutritional and cultural status, is also symbolically linked with live animals, blood, slaughter, aggression, and violence [21]. Consequently, animal-based foods are often accompanied by strong negative emotions [62]. Psychological literature consistently links disgust with animal-based products, making it necessary to control for the potential influence of disgust in this study [32,63].

### 3.2. Experimental Sample and Design

We recruited participants for this experiment through the Credamo platform (a well-known intelligent professional research platform in China). In Experiment 1, a total of 200 questionnaires were distributed via the Credamo platform, which randomly pushed the survey to internet users. After excluding incomplete and invalid responses, 188 valid questionnaires were retained, resulting in a response rate of 94%. The final sample had a mean age of 32.22 years (SD = 8.57), including 87 males (46.3%) and 101 females (53.70%). Upon completion of the experiment, participants received compensation ranging from 5 to 8 RMB.

### 3.3. Experimental Procedure

**Figure 2:**
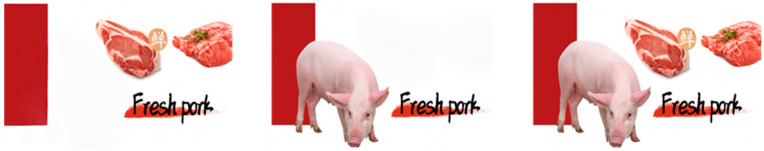
Stimulus materials of Experiment 1.

Experiment 1 used three types of pork advertisements collected online. Based on the research objectives, Photoshop was used to modify the stimuli to match the three designated advertising image types. Apart from the focal elements, the three stimuli were identical in textual descriptions and background design. Pork brand labels and other factors that could influence purchase decisions were uniformly removed to avoid confounding effects. Participants were randomly assigned to one of the three advertising groups: meat-typical, animal-typical, or composite. They were instructed to imagine the following scenario: “Pork is a staple food in daily life. Suppose you intend to purchase pork at a nearby fresh food supermarket. When you approach the pork counter, you see the following billboard displayed above it.” In the meat-typical group, the stimulus focused on pork slices; in the animal-typical group, it highlighted live pigs; and in the composite group, both pork slices and live pigs were featured. The specific stimuli are shown in Figure 2.

After viewing the stimulus materials, participants completed measures of purchase intention, guilt, disgust, and demographic variables. Purchase intention was measured using a scale adapted from Zeithaml’s (1988) purchase intention questionnaire, consisting of three items [64]. Guilt was measured with a scale adapted from Antonetti and Maklan’s guilt questionnaire [65], also consisting of three items. Disgust was measured with a single-item scale based on the method of Wang Haizhong et al. [66]. All items were rated on a seven-point Likert scale (1 = strongly disagree, 7 = strongly agree). The measurement items are presented in Appendix A. The Cronbach’s α coefficients for purchase intention and guilt were 0.90 and 0.88, respectively, indicating high reliability.

### 3.4. Results

**Figure 3:**
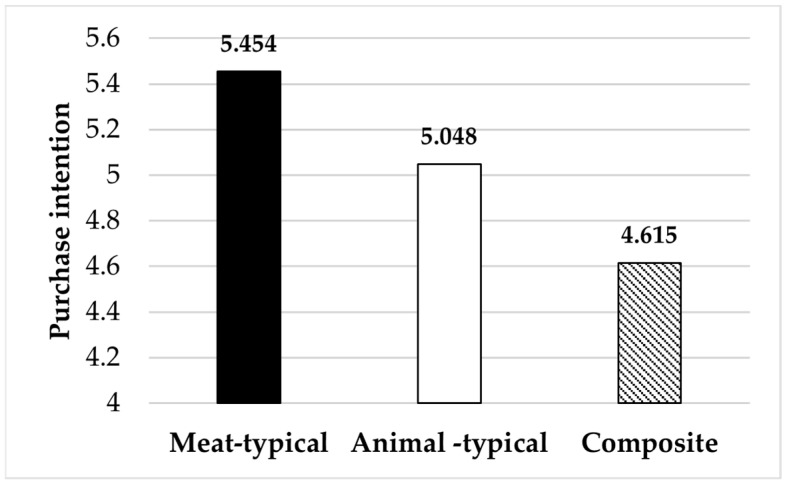
The impact of advertising image types on purchase intention.

Main effect: An analysis of variance (ANOVA) was conducted with advertising image type as the independent variable and purchase intention as the dependent variable. The results showed significant differences in purchase intention across the different advertising image types (F (2, 185) = 8.98, *p* = 0.000, η^2^ = 0.089). Specifically, purchase intention induced by meat-typical advertising images was significantly higher than that induced by animal-typical images (M_meat_ = 5.45 > M_animal_ = 5.05, F (1, 187) = 4.28, *p* = 0.043, η^2^ = 0.026); and purchase intention triggered by animal-typical images was significantly higher than that triggered by composite images (M_animal_ = 5.05 > M_composite_ = 4.61, F (1, 187) = 4.28, *p* = 0.029, η^2^ = 0.026). Additionally, purchase intention induced by meat-typical images was also significantly higher than that induced by composite images (M_meat_ = 5.45 > M_composite_ = 4.61, F (1, 187) = 8.98, *p* = 0.000, η^2^ = 0.053). These results suggest that, in pork advertising, meat-typical images (fresh meat slices) are the most effective, followed by animal-typical images (live pigs), and composite images are the least effective. In summary, Hypotheses 1 and 2 were supported (see Figure 3).

Mediating Effect of Guilt: First, Bootstrap Model 4 was used to examine the mediating roles of guilt and disgust in the relationship between advertising image types and purchase intention. The results indicated that the mediating effect of disgust was not significant, ruling out its potential explanatory role. As a result, subsequent analyses focused solely on the mediating effect of guilt. Advertising images were categorized into meat-typical, animal-typical, and composite types. Meat-typical images were set as the reference category, and two dummy variables were created: D1 for animal-typical images and D2 for composite images. Mediation analysis was conducted using D1 and D2 as independent variables, guilt as the mediator, and purchase intention as the dependent variable, incorporating the mediation model (Model 4, bootstrapping 5000 times). Following the approach of Fang et al. for mediation analysis with multicategorical independent variables, an overall mediation analysis was performed first [67]. The results showed that the overall total effect was significant (F (2, 185) = 8.980, *p* < 0.001), indicating that the two relative total effects were not all zero; the overall direct effect was also significant (F (2, 184) = 5.217, *p* < 0.001), suggesting that the two relative direct effects were not all zero. Furthermore, the two relative indirect effects were not both zero (95% CI = [−0.2453, −0.0321]. This result suggested the necessity of conducting further relative mediation analyses.

Further relative mediation analysis showed that, with meat-typical advertising images as the reference category, the relative mediation effect of animal-typical versus meat-typical was −0.090 (SE = 0.054, 95% CI = [−0.2137, −0.0064]). The confidence interval does not include 0, indicating that the mediating effect is significant (a_1_ = 0.546, b = −0.164, a_1_b = −0.090). Specifically, animal-typical advertising images significantly increased guilt by 0.546 compared with meat-typical images (a_1_ = 0.546), which in turn reduced purchase intention by 0.164 (b = −0.164). The relative direct effect was not significant (c′_1_ = −0.316, *p* = 0.120), suggesting that, after accounting for the mediation, the difference between animal-typical and meat-typical advertising on purchase intention was not significant. The relative total effect was significant (c_1_ = −0.406, *p* < 0.05), with the relative mediation effect (a_1_b) accounting for 22.2% of the total effect (0.090/0.406).

**Figure 4:**
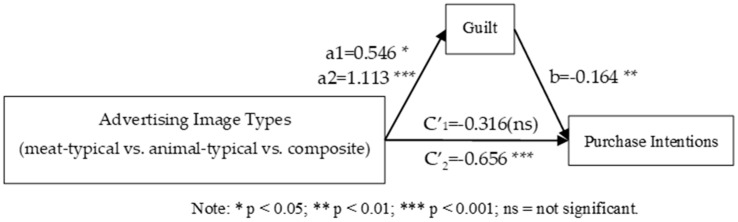
Mediating effect of guilt.

Similarly, using meat-typical advertising images as the reference category, the relative mediation effect of composite versus meat-typical was −0.1825 (SE = 0.076, 95% CI = [−0.3488, −0.0551]), which did not include zero, indicating that the mediating effect is significant (a_2_ = 1.113, b = −0.164, a_2_b = −0.183). The results indicated that composite advertising images significantly increased guilt by 1.113 compared with meat-typical images (a_2_ = 1.113), thereby reducing purchase intention by 0.164 (b = −0.164). In addition, the relative direct effect was significant (c′_2_ = −0.656, *p* < 0.01), suggesting that even after excluding the mediation, composite advertising images still reduced purchase intention by 0.656 compared with meat-typical images. The relative total effect was significant (c_2_ = −0.839, *p* < 0.001), with the relative mediation effect (a_2_b) accounting for 21.8% of the total effect (0.183/0.839). In summary, the findings confirm that guilt mediates the relationship between advertising image type and consumer purchase intention, thereby supporting Hypothesis 3 (see Figure 4).

### 3.5. Discussion

Experiment 1 examined the effect of advertising image types on consumers’ purchase intentions, confirmed guilt as a mediator, and excluded disgust as an alternative explanation. The results indicate that the three types of advertising images have significantly different effects on consumers’ purchase intention. Specifically, animal-typical images were the least effective, followed by composite images, while meat-typical images elicited the strongest purchase intentions. These differences are explained by the varying levels of guilt evoked by different image types: animal-typical and composite images elicited stronger feelings of guilt than meat-typical images, which in turn reduced consumers’ purchase intentions.

Despite strong empirical support for Hypotheses H1, H2, and H3, several limitations should be acknowledged. First, the study used fresh pork as the experimental stimulus. As pork is a staple food, participants’ familiarity and preference for it may have influenced their responses, limiting the generalizability of the findings. Future research should consider a broader range of fresh agricultural products to enhance the robustness and external validity of the findings. Second, the conclusions of Experiment 1 may be subject to boundary conditions. Further studies are needed to examine additional moderating factors that may influence the relationship between advertising image types, guilt, and purchase intentions.

## 4. Experiment 2: The Moderating Role of Self-Construal in the Relationship Between Advertising Image Types and Purchase Intentions

### 4.1. Experimental Purpose

Experiment 2 adopted a 3 (advertising image type: meat-typical vs. animal-typical vs. composite) × 2 (self-construal: interdependent vs. independent) between-subjects design. This study sought to investigate the moderating influence of self-construal on the relationship between advertising image types and consumer purchase intentions, to further validate the mediating effect of guilt, and to eliminate the alternative explanation of disgust. Furthermore, to augment the external validity and robustness of the findings, Experiment 2 used beef as the stimulus material, thereby evaluating the applicability of the conclusions in various fresh meat contexts.

### 4.2. Experimental Sample and Design

In Experiment 2, a total of 540 participants were randomly recruited via the Credamo platform. After excluding incomplete questionnaires and responses that failed the attention checks, 521 valid cases were retained, resulting in a response rate of 96%. The valid sample had an average age of 29.76 years (SD = 7.68), with 215 males (41.3%) and 306 females (58.7%). Participants received compensation of 5–8 RMB upon completing the experiment.

### 4.3. Experimental Procedure

In Experiment 2, the beef ads from the Internet were processed into the three types of advertising images in the study. Self-construal was manipulated using priming materials adapted from Ma et al. [59]. Participants were instructed to imagine themselves competing in a badminton tournament either as an individual player (independent self-construal) or as a team representative (interdependent self-construal), thereby activating different self-construal orientations. The effectiveness of this manipulation has been validated in previous studies [68]. Specifically, participants in the independent self-construal condition were asked to read the following priming material:

“You are participating in a badminton championship and have reached the finals. It is 3:32 p.m., and the sunlight shines on you. At this moment, you are the center of the world. You tell yourself: This is my battle; this is my opportunity. Regardless of winning or losing, I will prove my worth to myself.”

In the interdependent self-construal condition, participants were asked to read the following priming material:

“You are participating in a badminton championship, and you will represent your team in the finals. It is 3:32 p.m., and the sunlight shines on you while your coach and teammates cheer for you. You tell yourself: This is our team’s battle; this is our team’s opportunity. Regardless of winning or losing, I will prove our value to my team.”

Experiment 2 used a 3 (advertising image type: meat-typical vs. animal-typical vs. composite) × 2 (self-construal: independent vs. interdependent) between-subjects design, with participants randomly assigned to one of six groups. All participants first read the self-construal priming materials and completed the corresponding self-construal measurement scale. Participants were then asked to imagine the following scenario:

“Beef is a staple meat in daily life. Suppose you plan to purchase beef at a nearby fresh food supermarket; when you enter the meat section, you see the advertisement displayed above the counter.”

**Figure 5:**
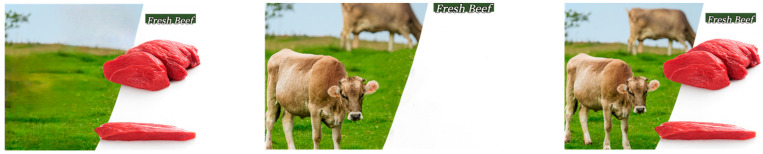
Stimulus materials of Experiment 2.

In the meat-typical condition, participants viewed an advertisement featuring fresh beef slices; in the animal-typical condition, the advertisement depicted a live cow; and in the composite condition, the advertisement presented both fresh beef slices and a live cow. Apart from the core stimulus differences, the text and background were identical across all advertisement images. The specific stimuli are shown in Figure 5.

Participants then completed measures of purchase intention, guilt, disgust, and demographic variables. Purchase intention was measured using a scale adapted from Zeithaml (1988) [66], consisting of three items; see Appendix A. Guilt and disgust were measured using the same scales as in Experiment 1.

Given that all participants were Chinese consumers, the self-construal scale was adapted from the Chinese version translated by Singelis (1994) and Wang Yuhao [69,70], comprising 14 items, with seven measuring independent self-construal and seven measuring interdependent self-construal (see Appendix A and Appendix B). All items were rated on a seven-point Likert scale (1 = strongly disagree, 7 = strongly agree). The results showed that the Cronbach’s α coefficients for purchase intention and guilt were 0.90, 0.88, respectively, indicating high reliability of the scales. We conducted a CFA to evaluate the factorial validity of the Chinese self-construal scale. The hypothesized two-factor model (independent vs. interdependent) showed a marginally acceptable fit to the data, χ^2^ (76) = 333.19, χ^2^/df = 4.38, RMSEA = 0.081 (90% CI [0.072, 0.090]), CFI = 0.889, TLI = 0.867, SRMR = 0.056. All standardized factor loadings were significant (*p* < 0.001). The overall Cronbach’s α = 0.72, indicating adequate internal consistency (see Appendix B).

### 4.4. Results

**Figure 6:**
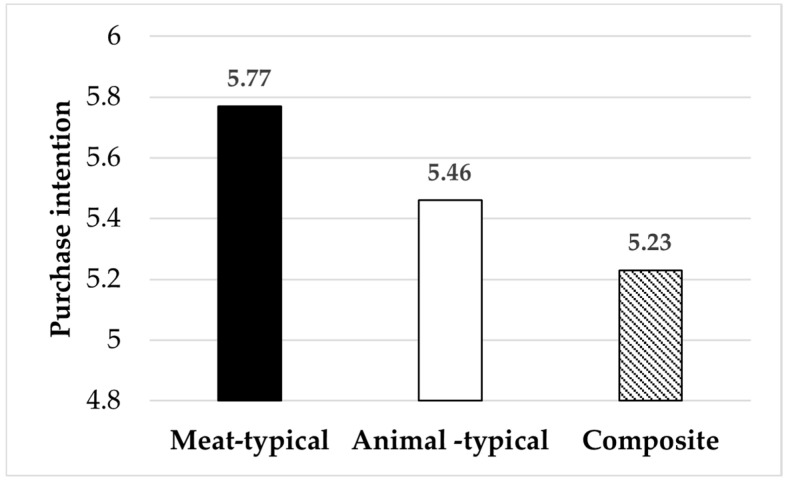
The impact of advertising image types on purchase intention.

Main effect: The results of the ANOVA indicated that different advertising image types had a significant effect on purchase intention (F (2, 518) = 12.39, *p* = 0.000, η^2^ = 0.046). Specifically, purchase intention elicited by meat-typical images was significantly higher than that elicited by animal-typical images (M_meat_ = 5.77 > M_animal_ = 5.46, F (1, 518) = 8.41, *p* = 0.004, η^2^ = 0.016). Purchase intention elicited by animal-typical images was significantly higher than that elicited by composite images (M_animal_ = 5.46 > M_composite_ = 5.23, F (1, 518) = 4.29, *p* = 0.039, η^2^ = 0.045). Purchase intention elicited by meat-typical images was also significantly higher than that elicited by composite images (M_meat_ = 5.77 > M_composite_ = 5.23, F (2, 518) = 24.50, *p* = 0.000, η^2^ = 0.016). In summary, for pork advertisements, images featuring meat slices yielded the most favorable sales outcomes, followed by live pig images, while composite images produced the least effective outcomes. Therefore, Hypotheses 1 and 2 were once again supported (see Figure 6).

Mediating Effect of Guilt: Consistent with Experiment 1, the mediation analysis was conducted using PROCESS Model 4 with 5000 bootstrap resamples. The mediating effect of disgust was not significant, and thus its alternative explanatory role was excluded. The overall mediation analysis showed a significant total effect (F (2, 515) = 12.39, *p* < 0.001), indicating that at least one of the two relative total effects was non-zero. The overall direct effect test was also significant (F (2, 517) = 7.86, *p* < 0.001), indicating that the two relative direct effects were not all zero. Furthermore, the two relative mediation effects were not both zero (95% CI = [−0.2453, −0.0321]). Therefore, it is necessary to conduct further relative mediation analyses.

The results of the relative mediation analysis indicated that, with meat-typical images as the reference group, the relative mediation effect of animal-typical versus meat-typical was −0.074 (SE = 0.034, 95% CI = [−0.1581, −0.0191]). The results confirmed a significant mediation effect (a_1_ = 0.355, b = −0.209, a_1_b = −0.074). Specifically, animal-typical advertising images elicited 0.355 higher levels of guilt compared to meat-typical images (a_1_ = 0.355), which in turn reduced purchase intention by 0.209 (b = −0.209). The relative direct effect was significant (c′_1_ = −0.235, *p* < 0.05), indicating that after controlling for the mediating role of guilt, animal-typical images still significantly reduced purchase intention compared with meat-typical images, by 0.235. The relative total effect was significant (c_1_ = −0.309, *p* < 0.01), and the relative mediation effect (a_1_b) accounted for 23.9% of the total effect (0.074/0.309).

**Figure 7:**
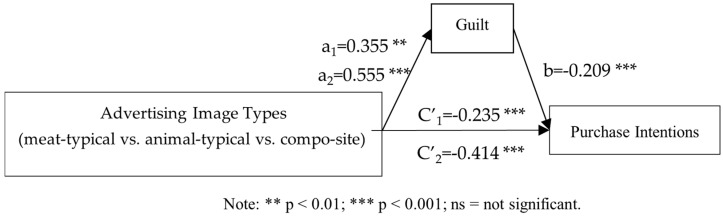
Mediating effect of guilt.

Similarly, with meat-typical images as the reference group, the relative mediation effect for composite versus meat-typical images was −0.116 (SE = 0.042, 95% CI = [−0.2150, −0.0485]), which did not include zero, indicating a significant mediation effect (a_2_ = 0.555, b = −0.209, a_2_b = −0.116). Specifically, composite advertising images elicited 0.555 higher levels of guilt than meat-typical images (a_2_ = 0.555), which in turn reduced purchase intention by 0.209 (b = −0.209). The relative direct effect was significant (c′_2_ = −0.414, *p* < 0.001), suggesting that even after accounting for the mediating role of guilt, composite images still reduced purchase intention by 0.414 compared to meat-typical images. The relative total effect was also significant (c_2_ = −0.530, *p* < 0.001), with the relative mediation effect (a_2_b) accounting for 21.9% of the total effect (0.116/0.530). Collectively, these findings confirm that guilt mediates the relationship between advertising image types and purchase intention, thereby providing further support for Hypothesis H3 (see Figure 7).

Manipulation Check: To assess the effectiveness of the self-construal manipulation, we first calculated the mean score of the seven items measuring independent self-construal and the mean score of the seven items measuring interdependent self-construal. Independent-sample *t*-tests revealed significant differences between the two groups. Specifically, participants in the interdependent condition reported significantly higher interdependent self-construal scores than those in the independent condition (M_independent_ = 4.86 < M_interdependent_ = 5.37, t = 6.44, *p* = 0.001). Conversely, participants in the independent condition reported significantly higher independent self-construal scores than those in the interdependent condition (M_independent_ = 5.49 > M_interdependent_ = 4.97, t = −6.49, *p* = 0.000). The results indicate that the manipulation of self-construal was successful.

We constructed a self-construal difference score (interdependent score minus independent score) and used the median split (−0.143) to classify participants. Participants with scores below the median were classified as having an independent self-construal, whereas those with scores at or above the median were classified as having an interdependent self-construal. An additional *t*-test confirmed that the two groups differed significantly in the self-construal difference score (t = −20.92, *p* < 0.001), supporting the validity of this categorization for subsequent analyses.

The dummy coding approach consistent with Experiment 1 was employed. Purchase intention served as the dependent variable, D1 and D2 as independent variables, guilt as the mediator, and self-construal as the moderator. Following the moderated mediation framework for categorical variables proposed by Fang et al. [71], PROCESS Model 7 (SPSS 4.0) was applied to examine the first-stage moderated mediation effect using 5000 bootstrap resamples.

Moderated Mediation Analysis: When self-construal was independent (Z = 0), with meat-typical images as the reference, the relative indirect effect of animal-typical advertising images was a_1_b_1_ = −0.049 (SE = 0.047, 95% CI = [−0.1474, 0.0305]). The confidence interval includes zero, indicating that the relative indirect effect was not significant. When self-construal was interdependent (Z = 1), compared to meat-typical images, the relative indirect effect of animal-typical images was (a_1_ + a_4_) b_1_ = −0.095 (SE = 0.045, 95% CI = [−0.1933, −0.0201]), and the confidence interval did not include zero, indicating a significant relative indirect effect. The difference between the interdependent and independent indirect effects was (a_1_ + a_4_) b_1_ − a_1_b_1_ = −0.047 (SE = 0.059, 95% CI = [−0.1664, −0.0645]), and this confidence interval included zero. Therefore, we can conclude that self-construal did not moderate the relative indirect effect of animal-typical compared to meat-typical advertising images.

**Figure 8:**
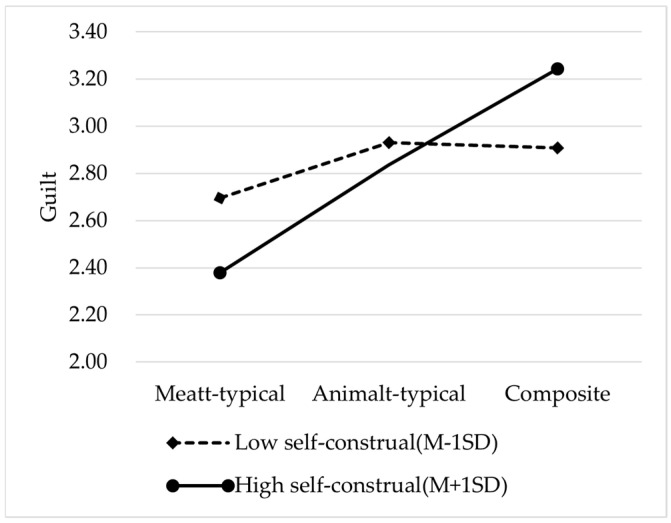
The moderating role of self-construal.

When self-construal was independent (Z = 0), with meat-typical advertising images as the reference, the relative indirect effect of composite advertising images was a_2_b_1_ = −0.038 (SE = 0.044, 95% CI = [−0.1441, −0.0329]), indicating that the relative indirect effect was not significant. When self-construal was interdependent (Z = 1), with meat-typical advertising images as the reference, the relative indirect effect of composite advertising images was (a_2_ + a_5_) b_1_ = −0.179 (SE = 0.059, 95% CI = [−0.3117, −0.0794]), and the confidence interval did not include zero, indicating that the relative indirect effect was significant. The difference between interdependent and independent indirect effects was (a_2_ + a_5_) b_1_ − a_2_b_1_ = −0.136 (SE = 0.064, 95% CI = [−0.2744, −0.0222]), and the confidence interval did not include zero. Therefore, it can be concluded that the relative indirect effect of composite advertising images (compared with meat-typical images) was moderated by self-construal. The results suggest that self-construal moderated the relationship between composite advertising images and purchase intention via guilt, thus supporting Hypothesis 4 (see Figure 8).

### 4.5. Discussion

Experiment 2 used beef as the stimulus to replicate the findings of Experiment 1 and further explore the moderating role of self-construal. Specifically, meat-typical advertising images were most effective in promoting consumers’ purchase of fresh agricultural products, while composite advertising images were least effective, as the guilt elicited by meat-typical images was lower than that triggered by animal-typical and composite images. Additionally, Experiment 2 found that for consumers with an interdependent self-construal, composite advertising images evoked stronger feelings of guilt, thereby reducing their marketing effectiveness.

Although Experiment 2 extended the boundary conditions of the findings from Experiment 1, several limitations remain. First, while Experiment 2 used beef as the stimulus, different types of meat may elicit distinct moral emotions in consumers, potentially limiting the generalizability of the findings to other meat categories. Second, as all participants were Chinese consumers, cultural differences may influence self-construal and guilt responses. Therefore, the cross-cultural generalizability of the findings requires further investigation in future research.

## 5. General Discussion

This study, adopting a negative emotion perspective, conducted two experiments to investigate the effects of advertising image types in the fresh food market on consumers’ purchase intentions, as well as the underlying mechanisms and boundary conditions. The results demonstrate that different types of advertising images (meat-typical, animal-typical, and composite) exert significantly different impacts on purchase intentions. Specifically, meat-typical images are more effective than animal-typical images in stimulating consumer purchases, while animal-typical images outperform composite images.

The underlying mechanism is that different types of pictures trigger different degrees of guilt among consumers. The results of the relative mediation effect analysis show that, with the typical meat picture as the reference level, the typical animal advertising image significantly increased the sense of guilt compared with the typical meat advertising image, thus reducing the willingness to buy. Similarly, compared with the typical meat picture, the composite picture triggered a higher sense of guilt, resulting in a lower willingness to buy.

In addition, the guilt triggered by the type of advertising image is significantly different under different levels of self-construal. Specifically, for consumers with a high level of interdependent self-construal, composite ads lead to higher guilt feelings and thus the negative impact on purchase intention is more obvious. These findings enrich the literature on visual advertising strategies by highlighting the critical role of guilt as a psychological mechanism and by identifying self-construal as a critical boundary condition shaping consumer responses to meat-related advertising.

Beyond the specific context of meat advertising, these findings contribute to a broader understanding of how moral emotions influence consumer decision-making. The present study demonstrates that guilt, as a self-conscious moral emotion, mediates the effect of product-related visual cues on purchase intention. This mechanism may also apply to other consumption contexts involving moral conflict, such as environmentally harmful products, animal-tested cosmetics, or unsustainable fashion, where consumers experience tension between personal preferences and ethical concerns [72,73]. These results offer theoretical insights into emotion-driven and morality-based consumer behavior.

### 5.1. Theoretical Contributions

From the perspective of consumers’ self-conscious emotions, this study employed experimental methods to examine the impact of advertising image types on consumers’ purchase intentions, while exploring the mediating role of guilt and the moderating role of self-construal. The theoretical contributions of this research are mainly reflected in the following aspects:

First, it enriches the literature on purchase intentions for fresh meat within the context of Chinese culture. Previous studies have demonstrated that factors such as pork appearance, quality concerns, and traceability characteristics significantly influence consumers’ purchase intentions [74,75,76]. Moreover, prior research has shown that the use of images in marketing materials can influence many important consumer outcome variables, including advertising and brand attitudes [77], information processing strategies [78], emotional responses [79], product inferences [80], and consumption levels [10]. This study classified pork advertising images into three categories, further examined their effects on consumers’ purchase intentions, and employed the framework of self-conscious emotions to reveal the mediating mechanism underlying consumers’ pork purchase intentions.

Second, it identifies the mediating role of consumer guilt in the relationship between types of pork advertising images and purchase intentions. The core focus of this study lies in uncovering the underlying mechanisms through which different advertising images differentially influence purchase intentions. Therefore, this study introduced consumer guilt and constructed a theoretical mechanism explaining the influence of advertising image types on purchase intentions, thereby addressing the gap in prior research that primarily relied on positive attitudes to explain consumer intentions and offering a novel perspective on how advertising images shape purchasing behavior.

Furthermore, the study reveals that composite advertising images are the least effective in marketing and that self-construal moderates the extent to which guilt mediates the influence of advertising image types, thereby deepening our understanding of when the effects of advertising images are most pronounced. Existing research on meat consumption has primarily examined moderating factors from the perspective of animal welfare and consumer choices, such as humane treatment of animals and consumers’ commitment to meat consumption [16], but relatively few studies have explored potential boundary effects from the perspective of individual consumer traits. This study not only highlights the negative effects of advertising images but also identifies the boundary conditions under which these effects occur, thereby enriching and advancing the body of research on advertising imagery.

### 5.2. Practical Contributions

This study, from the novel perspective of negative emotions, reveals how different types of meat advertising images influence consumers’ purchase intentions and offers actionable insights for corporate marketing practices.

First, firms should carefully consider the visual framing of meat advertisements. Although composite advertising images combine both animal- and meat-typical elements to convey product safety and naturalness, they may inadvertently activate consumers’ moral concerns about animal harm. To avoid this, firms are advised to conduct pre-tests of consumer emotional responses to advertising visuals and to use meat-typical images with bright, appetizing colors when the goal is to enhance purchase intention and emphasize product quality.

Second, when companies seek to differentiate their brands through composite advertisements that convey more comprehensive meat-related information, they should take measures to reduce consumer guilt associated with the “meat paradox.” This can be achieved by integrating clear welfare or traceability cues—such as certified farm identifiers, humane-treatment labels, or transparent supply-chain information—within the advertisement design. These cues can help neutralize guilt responses, strengthen perceptions of product integrity, and enhance consumer trust [81,82]. Additionally, price-based or utilitarian promotions (e.g., discounts or value bundles) may redirect consumers’ attention toward tangible benefits, thereby mitigating guilt and improving purchase intentions.

Finally, this research highlights that consumer self-construal moderates emotional responses to meat advertising. Firms should therefore adopt segment-specific communication strategies. For markets dominated by interdependent consumers, advertising should emphasize collective benefits—such as community health, food safety, or responsible production—to align with social values. For more independent consumer groups, messages emphasizing personal enjoyment, autonomy, and individual choice may be more persuasive. Overall, incorporating emotion-sensitive and culture-aware advertising strategies can substantially improve the effectiveness of marketing communications for fresh meat products.

### 5.3. Research Limitations and Future Work

There are some limitations to this study that future research should address. First, the use of static images as experimental stimuli may limit ecological validity, as video advertisements (now common in marketing) may elicit stronger and more immediate self-conscious emotional responses. Future research should consider using more diverse and dynamic stimuli. Second, the experimental design of this study is based on a survey of Chinese online consumers, which limits its generalizability due to the absence of cross-national samples and field experimental data. Future research should incorporate cross-national samples and randomized market surveys to enhance the generalizability of the findings by diversifying sample types and integrating real-world consumption data. Finally, this research primarily examined the boundary conditions of composite advertising images and lacked the discussion of possible boundary conditions such as animal welfare and traceability clues, in real advertisements. Future work could extend these boundaries to enrich the applicability and robustness of the conclusions.

## Data Availability

First, the data in this study include self-reported personal information from consumers. During the research process, participants were explicitly informed that their personal data would be securely stored and managed by the research team. In addition, the dataset involves ongoing and unpublished research conducted by the team; therefore, the data are not publicly available at this stage.

## Appendix A. Summary of Measurement Items

**Table:**

Construct	Measurement Items (Seven-Point Scales)	Reliability
Purchasing intention	I think I would purchase the pork from this counter. I would be willing to recommend the pork from this counter to others. The next time I buy pork, I will consider purchasing it from this counter.	Experiment 1
Purchasing intention	I think I would purchase the beef from this counter. I would be willing to recommend the beef from this counter to others. The next time I buy beef, I will consider purchasing it from this counter.	Experiment 2
Guilt	This advertisement makes me feel sympathy. This advertisement makes me feel guilty. This advertisement makes me feel remorse.	Experiment 1 Experiment 2
Disgust	I feel disgusted by this advertisement.	Experiment 1 Experiment 2

## Appendix B. Results of Confirmatory Factor Analysis (CFA)

**Table:**

Measurement Items	Factor	Standardized Loading	*p*-Value
The happiness of my team members is important to me.	Interdependent Self-Construal	0.740	<0.001
It is important to maintain harmony within the team.		0.698	<0.001
My happiness largely depends on the happiness of those around me.		0.736	<0.001
If my family disapproves, I am willing to give up an activity I really enjoy.		0.584	<0.001
Even if I strongly disagree with group members, I try to avoid conflict.		0.580	<0.001
If they need me, I would stay in a group even if I do not like it.		0.584	<0.001
Respecting the group’s decision is important to me.		0.536	<0.001
My personal identity is independent of others, and this is very important to me.	Independent Self-Construal	0.760	<0.001
I like to be different from others in many ways.		0.689	<0.001
I am unique.		0.555	<0.001
I would rather say “no” directly to others than risk being misunderstood.		0.458	<0.001
I enjoy working in situations where I am in competition with others.		0.507	<0.001
Competition is the law of nature.		0.604	<0.001
When discussing with others, I prefer to be straightforward.		0.802	<0.001
